# Review on Performance of *Aspergillus* and *Penicillium* Species in Biodegradation of Organochlorine and Organophosphorus Pesticides

**DOI:** 10.3390/microorganisms11061485

**Published:** 2023-06-02

**Authors:** Peter Matúš, Pavol Littera, Bence Farkas, Martin Urík

**Affiliations:** Institute of Laboratory Research on Geomaterials, Faculty of Natural Sciences, Comenius University in Bratislava, Mlynská dolina, Ilkovičova 6, 84215 Bratislava, Slovakiabence.farkas@uniba.sk (B.F.); martin.urik@uniba.sk (M.U.)

**Keywords:** biotransformation, filamentous fungi, organochlorine, organophosphorus pesticides

## Abstract

The use of pesticides in agricultural practices raises concerns considering the toxic effects they generate in the environment; thus, their sustainable application in crop production remains a challenge. One of the frequently addressed issues regarding their application includes the development of a sustainable and ecofriendly approach for their degradation. Since the filamentous fungi can bioremediate various xenobiotics owing to their efficient and versatile enzymatic machinery, this review has addressed their performance in the biodegradation of organochlorine and organophosphorus pesticides. It is focused particularly on fungal strains belonging to the genera *Aspergillus* and *Penicillium*, since both are ubiquitous in the environment, and often abundant in soils contaminated with xenobiotics. Most of the recent reviews on microbial biodegradation of pesticides focus primarily on bacteria, and the soil filamentous fungi are mentioned only marginally there. Therefore, in this review, we have attempted to demonstrate and highlight the exceptional potential of aspergilli and penicillia in degrading the organochlorine and organophosphorus pesticides (e.g., endosulfan, lindane, chlorpyrifos, and methyl parathion). These biologically active xenobiotics have been degraded by fungi into various metabolites efficaciously, or these are completely mineralized within a few days. Since they have demonstrated high rates of degradation activity, as well as high tolerance to pesticides, most of the *Aspergillus* and *Penicillium* species strains listed in this review are excellent candidates for the remediation of pesticide-contaminated soils.

## 1. Introduction

The current agricultural practice is contingent on the use of various pesticides since there is an urgent need to enhance the crop production to supply the rapidly increasing food-demand [1]. Throughout the globe, approximately 2 million tons of pesticides are produced and utilized annually, and it was estimated that in 2020, the global pesticide usage was approximated to be 3.5 million tons [2]. The application of pesticides is apparently advantageous since it diminishes the crop infestations, and thus, limits the harvest losses and positively affects the crops quality [3]. However, due to their potent biological activity as toxins and owing to their extensive or injudicious application, the heavy soil treatment with pesticides can endanger the wildlife. The pesticides and their toxic degradation products can enter the plant tissues and build up in the food chain or remain in the soil and water environments and negatively affect the soil fertility and water quality [4]. Thus, the pesticides pose a significant risk to the environment and to human health. The alarming increase in the production and usage of pesticides triggered the notion to reduce the impact of pesticides and find sustainable alternative solutions to protect the crops [5].

Although the role of many pesticides in the environmental deterioration has not been adequately resolved, it is indisputable that they have adverse effects on various non-target organisms [6]. Thus, the research on advanced practices protecting wildlife, which highlights a more cautious use of synthetic agrochemicals, careful risk assessment, and licensing, is very much needed [7]. Therefore, the development of ecofriendly technologies focused on reducing the utilization of synthetic pesticides, especially those with high persistence in the environment, has been addressed [8,9]. More importantly, there is still an issue of developing a proper treatment technology for the remediation of pesticide-contaminated soils. This is a complex problem, since the areas with point-source contamination are usually agricultural soils whose properties should be maintained; thus, aggressive technologies should be omitted [10]. Among more recent and ecologically acceptable emerging technologies is bioremediation, which involves the utilization of indigenous microflora, adapted or genetically engineered microorganisms or their enzymes for the degradation and conversion of pesticides to another form via co-metabolism or mineralization [11,12].

Bhatt et al. [13] concluded that the microbial consortia are an essential biotechnological tool for a successful degradation of the recalcitrant pesticides in the natural (soil and water) environments. Engineering such a consortium in the form of biofilms is advantageous, but challenging, with high prospects in large-scale utilization [14]. However, most of the recent comprehensive reviews on the biodegradation of pesticides focus primarily on bacterial interaction with pesticides, while fungi are mentioned only marginally. Bacteria are the main microorganisms evaluated for pesticide degradation, since their catabolic metabolic pathways are far more understood in comparison to fungi, providing more opportunities for researchers to regulate and enhance their performance on molecular levels. Furthermore, bacteria are capable of utilizing pesticides as a sole source of carbon and nitrogen; thus, the research on pesticide utilization by fungi in biodegradation is scarce [15]. Still, in their recent commentary, Hyde et al. [16] highlighted that fungi possess the unparallel potential to degrade recalcitrant organic pollutants in the environment due to their ability to exude lignolytic extracellular enzymes and acidic metabolites. Fungal potential in the remediation of sites contaminated with recalcitrant, persistent, and toxic pollutants has been investigated for some decades [17]. Still, the implementation of laboratory procedures into successful field applications is rare [18], since the maintenance of fungal strains with high degradation activity in open purification systems in competition with indigenous microorganisms remains an unresolved problem [19].

The experimental studies on the ability of different fungal strains to accumulate and remove pesticides from the environment are mostly focused on (1) indigenous fungi growing at the contaminated sites, or (2) fungi with an enormous physiological capacity to degrade lignin [20], which is usually associated with the capability to biodegrade xenobiotics [21]. The latter depends primarily on the performance of non-specific peroxidase enzyme systems of white-rot fungi from a variety of basidiomycete orders, including strains of *Phanerochaete chrysosporium* and *Trametes versicolor* [22,23,24]. These orders are usually the primary object of experimental research on fungal biodegradation of pesticides. However, the former fungal group, which usually includes various non-white-rot fungi belonging to the Ascomycota and Zygomycota phylum, have also demonstrated great potential in the enzymatic transformation of environmental pollutants [25]. Recently, the review by Magnoli et al. [26] supported the notion that the experimental degradation of organochlorinated herbicide 2,4-dichlorophenoxyacetic acid is usually carried out by bacteria, and therefore, little is actually known about the fungal performance in pesticide’s biodegradation. Such a reviews are much needed for environmental mycologists since they may provide the basis for further exploration of the biotechnological potential of fungal enzymatic machinery. Thus, the present review outlines the fungal performance in the biodegradation of various organochlorine and organophosphorus pesticides (Table 1) to provide information that is essential for designing the strategy offsetting the negative impact of pesticides. Since among the Ascomycota phylum, the fungal species of the genera *Penicillium* and *Aspergillus* have shown the high richness and dominant abundance in soils highly polluted with chlorinated hydrocarbons [27], glyphosate [28], and soils treated with various herbicides [29], including atrazine and metolachlor [30], it inspired us to focus the presented review on the biodegradation of organochlorine and organophosphorus pesticides solely by isolates belonging to these common soil filamentous fungal genera.

## 2. Degradation of Organochlorine Pesticides by *Aspergillus* and *Penicillium* Species

**Figure 1:**
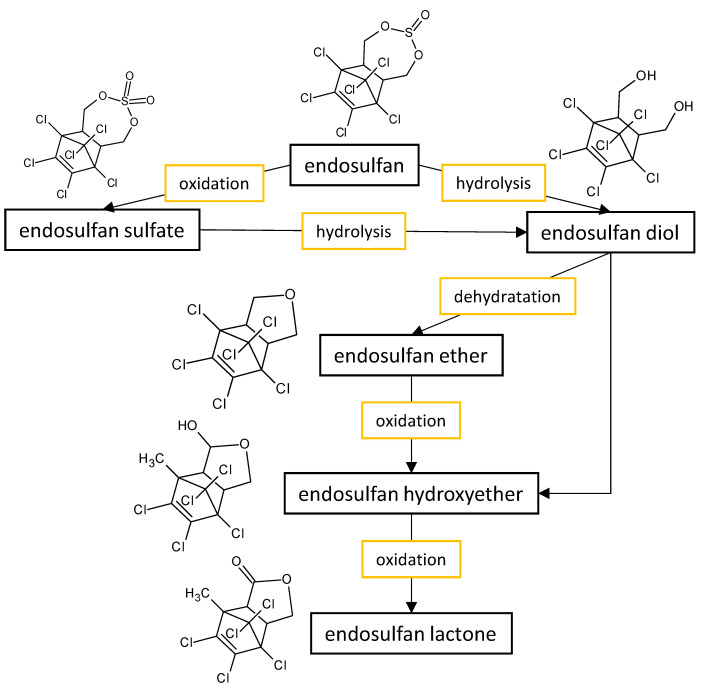
Proposed metabolic pathway for the degradation of endosulfan by *Aspergillus* and *Penicillium* fungal strains.

The fungal performance in the biodegradation of the most common organochlorine pesticides (endosulfan and lindane) in culture media, as well as the transformants or end products (Figure 1) formed by the fungi belonging to *Aspergillus* and *Penicillium* genera are listed in Table 2.

Romero-Aguilar et al. [31] isolated the filamentous fungus *Penicillium* sp. strain CHE 23 (99% sequence identity with *Penicillium expansum* and *Penicillium janthinellum*) from the activated sludge collected at the industrial wastewater treatment plant. It was capable of growing with endosulfan, a broad-spectrum cyclodieneorganochlorine insecticide (6,7,8,9,10,10-hexachloro-1,5,5a,6,9,9a-hexahydro-6,9-methano-2,3,4-benzo(e)dioxathiepin-3-oxide), as the sole carbon source. It performed well considering endosulfan biodegradation with almost 95% removal efficiency of its initial 56.7 mg·L^−1^ concentration in six days. Romero-Aguilar et al. [31] hypothesized that the fungal strain degraded or limited the generation of any hazardous endosulfan sulfate metabolites, since both acute and genotoxicity tests using earthworms (*Eisenia fetida*), a bioindicator organism, did not show any difference from endosulfan-free treatments. However, they carefully referred to this process as the removal or transformation/degradation, since no secondary metabolites have been analyzed or shown in the study. Unfortunately, there was no follow-up research addressing this issue, and thus, the authors did not provide any insight into the actual mechanism of pesticide removal.

Similarly, Bhalerao and Puranik [32] did not provide information on the exact mechanism of endosulfan biodegradation and only noted the process of mineralization (since the evolved CO_2_ was quantified), and the possibility of direct desulfurization of endosulfan sulfate by the fungus. In the study, they have used a technical grade endosulfan (350 mg·L^−1^) to estimate the presence of degradation products, including *α* and *β* endosulfan isomers, endosulfan sulfate, and endosulfan diol, during the cultivation of endosulfan-tolerant fungus *Aspergillus niger* on Czapek–Dox broth. They reported complete degradation of endosulfan by this common soil fungus as early as after 120 h, with *α* and *β* endosulfan disappearing from the culture medium after a day.

A rapid complete mineralization of 1.0 g·L^−1^ endosulfan within a week by the *A. niger* ARIFCC 1053 strain was reported by Tejomyee [33]. It was suggested that the degradation was initiated by the oxidation to endosulfan sulfate and its hydrolysis to endosulfan diol. Various intermediates were formed, including 1-chloro-1,3-propandiol and 2-chloro-hydroxyl-butanol. Furthermore, glyxal, formic, and protonated sulfurous acids were produced during endosulfan degradation that is likely to be ultimately converted to CO_2_, SO_2,_ and water in the environment. More importantly, less than 3% of endosulfan was degraded by abiotic reactions, which is negligible in comparison to the findings of the following studies.

Mukherjee and Gopal [34] studied the biodegradation of the more persistent *β* isomer of endosulfan by *A. niger*. The insecticide was dissipated by 98.6% at the end of the 15th day. No endosulfan sulfate was detected in the medium during cultivation, and, unfortunately, no other conversion products were measured since they were considered non-toxic by authors. Surprisingly, the fungus-free Czapek–Dox broth medium significantly decreased the *β* endosulfan content, with reported recovery approximating 22%. It suggests that some broth medium’s component is involved in or catalyzes the endosulfan transformation. However, this issue was not resolved even in the follow-up study [35]. There, the potato dextrose medium was capable of degrading almost 67% of endosulfan chemically, while the fungus *Aspergillus terreus* removed 91.5% after 15-day cultivation. Toxic endosulfan sulfate was detected in the presence of fungus only in small quantities in the middle of the cultivation, while no endosulfan diol was detected.

Similarly, Hussain et al. [36] did not observe the accumulation of endosulfan sulfate in the broth of non-sulfur medium inoculated with *Aspergillus terricola* and *A. terreus*, while endosulfan ether and diol have been identified as the main products of endosulfan metabolism. It is possible that these fungi strictly adopted hydrolytic pathways for biodegradation. Nevertheless, the 20% abiotic degradation of endosulfan within the 12-day incubation was also observed, which further increased with pH. However, after excluding the abiotic transformation, the degradation of both isomers by fungi ranged between 69.5% and 74.7% of the initial 100 mg·L^−1^ endosulfan concentration at the end of cultivation. The optimal conditions for biodegradation were at an initial broth pH of 6.0, incubation temperature of 30 °C, and dynamic cultivation (agitation).

The fungal culture dependent biodegradation of endosulfan by fungi in culture media remains controversial, since there, under specific conditions, the endosulfan can be abiotically degraded completely within two weeks. This was the case reported by Mukhtar et al. [37], who incubated 0.1% endosulfan in Czapek–Dox broth medium at 30 °C and 180 rpm. Still, the fungus *A. niger* accelerated the transformation of endosulfan to the extent that, after two days, lesser than 10% of remaining endosulfan was detected in the medium. Furthermore, the acidification of media to a pH of 4 and 5 before inoculation seems to be favorable for biodegradation kinetics. The positive effect of acidification was also reported by Ahmad [38], who suggested that the endosulfan diol is formed rapidly via hydrolysis, and indicated that fungal production of acidic metabolites favored the biologically induced degradation of endosulfan. This is slowly transformed into other intermediates, endosulfan ether, and endosulfan hydroxyether, which have a non-accumulative nature. Finally, endosulfan lactone was detected on GC-MS chromatograms, which was a unique product among the studied filamentous fungi. Ahmad [38] also reported that the endosulfan half-life was 17.3 days when incubated in the presence of *A. niger* for 35 days, and it followed the first-order reaction kinetics well. The other cultivated fungal strains of *Penicillium chrysogenum* and *Aspergillus flavus* were not as successful in biodegradation, since the values of calculated endosulfan half-life were 69.3 days and 34.4 days, respectively. The *A. niger* strain was capable of transforming 77% of endosulfan to its degradation products.

Small quantities of endosulfan lactone (2%) were also detected on the 6th day of endosulfan incubation (of an initial 100 mg·L^−1^ concentration) in the presence of *Aspergillus sydoni* [39]. It was identified along with the endosulfan ether (3.5%) and endosulfan sulfate (15%), which is most likely the main metabolite formed in broth culture. However, endosulfan lactone was a more prominent metabolite in the soil microcosm study. Although the fungus was capable of growing solely using endosulfan as the carbon source, the addition of sucrose decreased the degradation efficiency in broth culture, while it increased the transformation in the soil study. Still, fungus degraded α endosulfan by as much as 95% and 92.6% and *β* endosulfan by 97% and 77.5% within 18 and 60 days in broth culture and soil microcosm, respectively. Goswami et al. [39] also highlighted that both oxidative and hydrolytic pathways were utilized by the *A. sydoni* to degrade endosulfan.

Supreeth and Raju [45] reviewed the possible pathways of endosulfan biodegradation by filamentous fungi and came to the same conclusion—filamentous fungi are capable of utilizing endosulfan in both oxidative and hydrolytic pathways. While oxidative biotransformation results in the accumulation of endosulfan sulfate and endosulfan dimethylene, the hydrolytic pathway transformed endosulfan into endosulfan diol, ether, hydroxyether, and lactone.

Endosulfan degradation by *Aspergillus* species was also studied in soil microcosms by Silambarasan and Abraham [40]. There, the strain of *Aspergillus tamarii* JAS9 efficiently degraded *α* and *β* endosulfan isomers within 10 days of incubation and decreased the initial 300 mg·kg^−1^ concentration of *α* and *β* isomers by 50% after 2.3 and 3.5 days, respectively, according to first-order kinetics. No endosulfan sulfate was detected in the soil microcosm, while monitoring the metabolites in Czapek–Dox broth revealed its presence (no data were provided in the publication on this issue).

In a follow-up study, Abraham and Silambarasan [41] applied *A. tamarii* JAS9 as a part of a fungal consortium that included also strains of *Lasiodiplodia* sp. JAS12 and *Botryosphaeria laricina* JAS6. This time, the zero-order kinetics was suggested as the best fit for the endosulfan degradation by the fungal consortium in soil microcosm with the pesticide concentration decrease by 50% in 3.3 days.

Taşeli [42] investigated the capability of *Penicillium camemberti* to degrade lindane (gamma-hexachlorocyclohexane), an agricultural insecticide, under static and dynamic culture conditions. The 1 mM lindane was supplemented in the basal medium, and it served as a co-substrate. The addition of acetate (0.5 g·L^−1^) into culture media decreased the lindane conversion rate by the fungus from 70% to 57% within 10-day cultivation, presumably due to shifting to aerobic respiration. Thus, it can be hypothesized that the organic chlorine serves as an electron acceptor for the fungus. This is supported by the respirometric study of Taşeli and Gökçay [46], who highlighted that the organic chlorine could act as an electron acceptor for *P. camemberti*.

Along with the other three filamentous fungi, the strains of *Aspergillus flavus* and *A. niger* had the highest incidence in organochlorine-polluted sites and possessed a high ability to tolerate and degrade lindane [47]. Its initial 45 mg·kg^−1^ concentration was reduced by approximately 73.5% after 90-day incubation in the soil. However, the synergistic effect of guinea grass (*Megathyrsus maximus*) roots on active lindane dechlorination and transformations enhanced the degradation efficiency by up to 88.7%, depending on the amount of fungal inocula.

Kumaravel et al. [43] reported that the *Aspergillus fumigatus* was capable of degrading lindane completely during 5-day incubation on the Sabouraud dextrose medium. However, no information on initial lindane concentration or its degradation products was provided, despite the GC-MS chromatogram being displayed in the publication.

Birolli et al. [44] showed that the marine fungal strain *Penicillium miczynskii* was capable of biodegrading organochlorine pesticide dieldrin. Unfortunately, no intermediate organochlorine products of dieldrin were detected, even though fungi promoted its biotransformation by up to 90% within 14 days from its initial 50 mg·L^−1^ concentration. Interestingly, the hydrogen peroxide has been supplemented with dieldrin simultaneously into a five-day-old culture, which is supposed to facilitate biodegradation. This approach was promoted by Ortega et al. [48], who biodegraded dichlorodiphenyldichloroethane using the marine fungal strains of *Aspergillus sydowii* and *P*. *miczynskii*. Since the presence of peroxide increased the degradation kinetics, they suggested the involvement of peroxidases in dichlorodiphenyldichloroethane biotransformation, most likely in dehalogenation reactions [49].

## 3. Degradation of Organophosphorus Pesticides by *Aspergillus* and *Penicillium* Species

**Figure 2:**
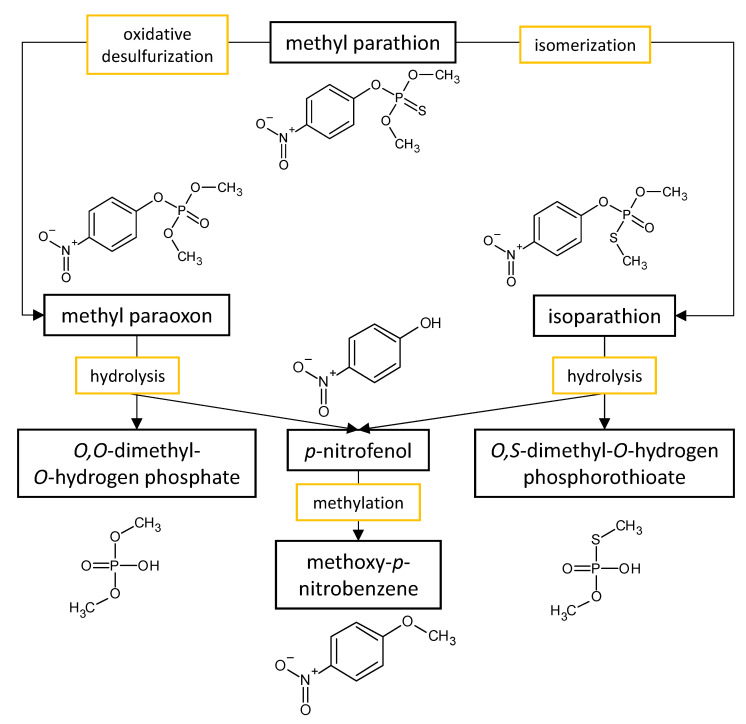
Proposed metabolic pathway for the degradation of methyl parathion by *Aspergillus* and *Penicillium* fungal strains.

**Figure 3:**
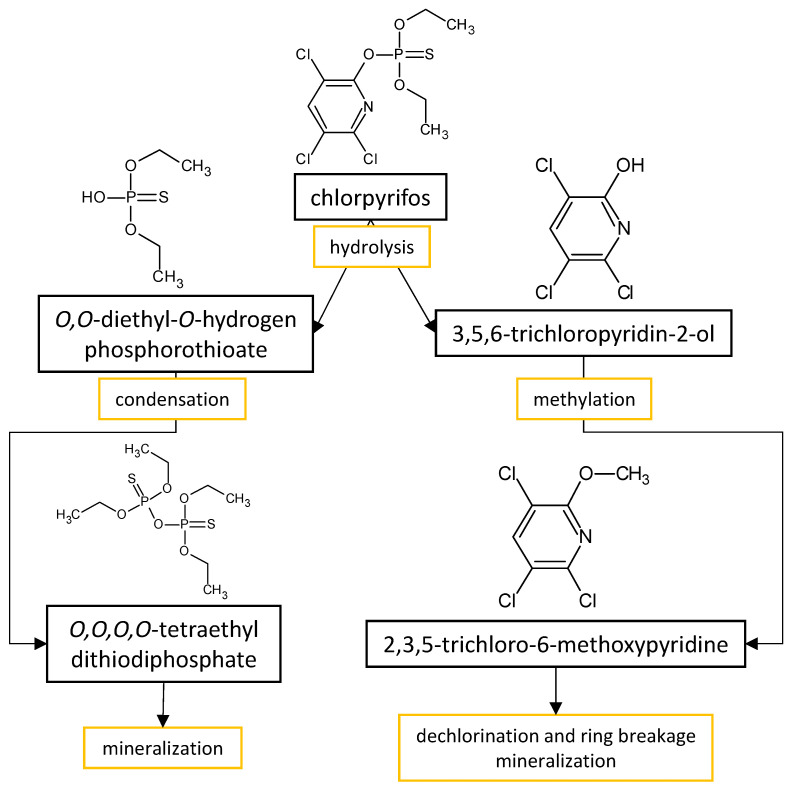
Proposed metabolic pathway for the degradation of chlorpyrifos by *Aspergillus* and *Penicillium* fungal strains.

The performances of strains belonging to *Aspergillus* and *Penicillium* genera in the biodegradation of organophosphorus pesticides in culture media are listed in Table 3. The summarized biodegradation pathways for methyl parathion and chlorpyrifos, the most studied organophosphorus pesticides, are depicted in Figure 2 and Figure 3, respectively.

Soares et al. [50] studied the biodegradation of organophosphorus pesticides, including chlorpyrifos (O,O-diethyl O-(3,5,6-trichloro-2-pyridyl phosphorothioate)), methyl parathion (O,O-dimethyl-O-(4-nitrophenyl)phosphorothioate), and profenofos (O-4-bromo-2-chlorophenyl O-ethyl S-propyl phosphorothioate) by the marine-derived fungal strain *A. sydowii* strain CBMAI 935 that was cultivated on 2% malt liquid culture media enriched with initial 50 mg·L^−1^ pesticide concentration. The fungal culture biodegraded chlorpyrifos, methyl parathion, and profenofos by 32%, 80%, and 52%, respectively, within 30 days. The authors have shown that the hydrolytic product 4-bromo-2-chlorophenol, which is considered the main metabolite of profenofos biodegradation pathway by fungi and bacteria, has been transformed by the *A. sydowii* fungus into 4-bromo-2-chloro-1-methoxybenzene, and it was likely degraded to smaller molecules thereafter. The metabolite *p*-nitrophenol that is the main and highly toxic product of methyl parathion transformation has been also shown to be biodegradable by this marine-derived fungus after being methylated [50]. The methylation helps to mitigate the toxic effects of the *p*-nitrophenol on fungus. The *A. sydowii* promotes excellent biodegradation of methyl parathion since using the pesticide as a sole carbon source reached the satisfactory biodegradation efficiency of 80% [51]. Alvarenga et al. [51] also noted that after being isomerized into isoparathion, and oxidized into a paraoxon, methyl parathion can be completely degraded in 20 days when an additional organic source is available to fungus. The authors suggest that the *A. sydowii* likely mineralized all the degradation products since, besides the low concentrations of *p*-nitrophenol, no other metabolites were found.

Marinho et al. [52] also hypothesized that the addition of glucose should improve the efficiency of *A. niger* to biodegrade methyl parathion in batch reactors. Since the presence of glucose increased the conversion rate, it proved indispensable for the efficient removal of methyl parathion by submerged culture of fungus. The initial reaction rate increased significantly after the addition of glucose in comparison to glucose-free treatment, with 43% removal efficiency in 30 days at an initial methyl parathion concentration of 24.9 mg·L^−1^. The first-order kinetic constant was calculated to be 0.162 h^−1^ in the presence of glucose while being only 0.063 h^−1^ in its absence.

It seems that the marine microorganisms are excellent sources for enzymes promoting the biodegradation of various xenobiotics, including organophosphates. This is also noted by Rodrigues et al. [53], who studied two marine-derived strains of *Penicillium citrinum*, isolated from ascidian *Didemnum ligulum*, for their capability to biodegrade the methyl parathion. After 20 days, its conversion in the presence of fungi was already completed. However, the control experiment revealed that 85% of pesticide was spontaneously degraded abiotically. This was facilitated most likely by the specific incubation conditions that promoted the alkaline hydrolysis of methyl parathion (pH 8 and 32 °C). Still, fungi could mineralize the main methyl parathion degradation metabolite *p*-nitrophenol, whose transformation was significantly promoted by the fungus (80%) in comparison to the abiotic control (30%). Rodrigues et al. [53] also suggested that it may have been used as a source of nutrients by the fungi and, thus, the *p*-nitrophenol was degraded or bioconjugated by them more efficiently.

Mohapatra et al. [54] developed a malathion-tolerant strain of *A. niger* that was immobilized in coir pith. It was applied in batch cultures to remove insecticides within five days. The highest rate of degradation was measured for malathion (~70%), followed by dimethoate (~68%), chlorpyrifos (~58%), and parathion (~54%). More importantly, the storage of immobilized fungus in coir pith up to 45 days did not alter the viability, and biological activity of fungus, as well as the removal efficiency of the tested pesticides. This can help to maintain fungus active before field application. It is also important to note that the autodegradation of the insecticides did not exceed 3.9% in control treatments.

Malathion, used as the sole carbon source, was also incubated in the presence of *A. flavus*, which resulted in the complete degradation of the pesticides in 36 days under optimized conditions [55]. Its transformation by the fungus has been favored in mineral media with pH 7 at 30 °C, and the malathion half-life was calculated to be 8.5 days. Thus, the fungal strain showed exceptional ability to accelerate malathion degradation, and since it was isolated from persistent organic pesticide-contaminated drainage, its natural resistance to xenobiotics should enhance its effectiveness in the remediation of malathion contaminated waters. The authors [55] also highlighted that the optimization of operating conditions is essential for the successful application of pesticide biodegradation by fungi, since avoiding the proper tuning of the process in the bioreactor may significantly decrease the rate of pesticide conversion. E.g., Yadav et al. [56] studied the kinetics of aerobic biodegradation of chlorpyrifos by a fungal isolate *Aspergillus* sp. F1 in the batch and continuous packed bed bioreactors to optimize the process parameters. The optimum values for batch degradation were 5.8 mg·L^−1^ of dissolved oxygen, 2.5 mg·L^−1^ wet weight of inoculum level, pH of 7.0, and a temperature of 28 °C. The 90% removal efficiency decreased when the initial chlorpyrifos concentration was over 300 mg·L^−1^, most likely due to inhibitory effects of transformants on the microbial population. The operating range for the continuous bioreactor was found to be in the range of 180 to 250 mg·L^−1^·d^−1^ with 89% removal efficiency at an inlet load of 180 mg·L^−1^·d^−1^ during operation of 45 days. There are also other attempts to optimize the biodegradation process. E.g., Anggreini et al. [57] utilized the Monod equation with the first-order kinetic model to calculate the degradation rate of chlorpyrifos by *A. fumigatus* at 0.45 day^−1^. The pesticide concentration decreased from an initial 50 mg·L^−1^ to 0.1 mg·L^−1^ within 9 days. Furthermore, the degradation rate was significantly affected by the initial dose of biomass. It increased from 79% to approximately 90% with increasing supplemented biomass weight in the range from 0.5 mg to 1.5 mg. In their previous report, Anggreini et al. [58] also concluded that the optimum temperature for the chlorpyrifos degradation by *A. fumigatus* was 25 °C. However, if optimized, the bacterial degradation capacities can still be superior, since the bacterial isolate of *Pseudomonas* Iso 1 outperformed the degradation by fungus *Aspergillus* sp. F1 in both the elimination and removal capacity in the same environment [56,67].

The successful strategy to further enhance the biodegradation capacities of filamentous fungi is most likely a selective adaptation of fungus on specific pesticide, or its isolation directly from the pesticide-exposed soils. The latter approach has been tested by Barberis et al. [60] who reported that two *Aspergillus oryzae* strains, AM 1 and AM 2, isolated from soils that have been exposed to pesticides in last decade, highly varied in the ability to degrade 20 mg·L^−1^ chlorpyrifos after 30 days of incubation. While AM 1 strain removed 50%, the AM 2 strain removed approximately 73%. Since there was a statistically significant and powerful positive correlation between the degradation efficiency and the growth rate, and *A. oryzae* AM 2’s mycelial growth was superior while having the shorter *lag* phase, it outperformed the biodegradation activity of *A. oryzae* AM 1 strain. Still, the higher tolerance does not directly imply the higher degradation efficiency.

The strategy of conditioning the fungus to selected pesticide has been applied by Silambarasan and Abraham [59] for the studying of biodegradation of chlorpyrifos by *Aspergillus terreus* strain JAS1, a chlorpyrifos-adapted strain, in soil and mineral. Both the pesticide and its major degradation product (3,5,6-trichloro-2-pyridinol) have been conversed by the fungus completely within two days in both the culture media and soil. Interestingly, the presence or absence of other nutrient sources in the soil did not affect the fungal performance. This indicates that the chlorpyrifos-degrading enzymes in *A. terreus* JAS1 are expressed even in the absence of readily available nutrients, and, thus, the fungus can be employed even in the environment poor in organic matter utilizing the chlorpyrifos as a sole carbon and energy source for microbial growth.

Abdel-Wareth and Abd El-Hamid [61] tested the ability of *Aspergillus viridinutans* and *Penicillium implicatum* cultivated on potato dextrose broth media to degrade pesticides. A 14-day incubation of 2.5 mg·L^−1^ chlorpyrifos in fungal presence allowed 100% successful transformation, while abiotic control showed only 18% degradation efficiency. However, the initial 20 mg·L^−1^ concentration significantly decreased the biodegradation performance of *A. viridinutans* and *P. implicatum* to approximately 45% and 16%, respectively. The control treatment decreased the concentration of chlorpyrifos by 13.5%. It is suggested that the observed concentration decrease in fungal-free treatments may be caused by pesticide aggregation with sugars or other molecules present in media, or by spontaneous hydrolysis, and non-biological oxidations and reductions. A similar issue has been reported by Alvarenga et al. [68] who noted significant 100% and 61% loss of 50 mg·L^−1^ chlorpyrifos in 2% malt extract and distilled water, respectively, within 30 days. Since they did not identify any degradation product of chlorpyrifos in the medium, they concluded that spontaneous hydrolysis is unlikely under these conditions, and aggregation with sugars or other molecules present in media more likely relates to the disappearance of chlorpyrifos. They also suggested that in distilled water, the loss due to volatilization is the most plausible hypothesis.

In some cases, this issue is addressed controversially. Despite the recorded 66.3% loss of chlorpyrifos in a fungal-free treatment, Mukherjee and Gopal [62] concluded that pesticide dissipation to 72.2% in the presence of *A. niger* by the end of 14-day cultivation signifies the role of fungus in chlorpyrifos degradation. Similarly, Abd-Alrahman and Mostafa [63] noted that the loss of chlorpyrifos from the medium, when incubated in presence of *P. citrinum*, was 26%, while the spontaneous abiotic degradation in fungal-free control reached 24.5%. Fortunately, the strains of *A. niger* and *A. oryzae* showed moderate, but promising biodegradation capacities of 64% and 50.8%, respectively. These values were significantly higher in comparison to abiotic degradation, highlighting the potency of *Aspergillus* species in chlorpyrifos-contaminated soils remediation.

Still, soil environment is complex, and components of soil solution can significantly limit the efficiency of enzymatic degradation of pesticides by fungi. Thus, Karas et al. [22] studied chlorpyrifos degradation by *A. niger* in a soil extract medium prepared according to Karpouzas et al. [69] to mimic fungal natural habitat. There, the rapid over 80% loss of its initial 10 mg·L^−1^ was observed in two days, followed by negligible changes and slow degradation thereafter until the end of 30-day cultivation. The metabolite 3,5,6-trichloro-2-pyridinol was formed gradually but did not exceed 1 mg·L^−1^.

Within various isolates of *Aspergillus* species, the strain of *A. terreus* was the most potent in organic phosphor and sulfur mineralization from organophosphorus insecticides, followed by *Aspergillus tamarii* and *A. niger* [64]. Organic phosphor mineralization by these fungi was highly correlated with extracellular protein content; thus, a direct relationship between insecticide degradation and extracellular protein excretion was suggested. Thus, the production of extracellular fungal phytases seems to be a viable method for organophosphorus pesticide degradation. This was successfully applied by Shah et al. [70], who collected phytases produced by *A. niger* NCIM 563 under submerged fermentation conditions and used them for in vitro degradation of monocrotophos, methyl parathion, and chlorpyrifos. The optimal enzyme conditions of pH 2.5 at 50 °C (100 IU; specific activity 53 IU·mg^−1^) allowed, after 2 h of exposure, the decrease in the relative area seen at the retention time of chlorpyrifos by 91%, in comparison to phytase-free controls. The application of higher units (250 IU) to degrade chlorpyrifos on the post-harvest chilies showed that 90% degradation was possible in 12 h.

Apart from methamidophos, the *Penicillium oxalicum* strain ZHJ6 was capable of degrading other organophosphorus pesticides, including folimat, phoxim, and glyphosate with glucose as a carbon source, while it could not degrade chlorpyrifos, phosdrin, trichlorphon, and dichlorvos [65]. The biodegradation process was optimized for mineral medium with pH 5 and 1% glucose, while incubated at 25 °C.

Fu et al. [66] characterized the process of glyphosate degradation by the fungus *A. oryzae*, where the aminomethylphosphonic acid is formed as the initial transformant, followed by its degradation into methylamine. The former conversion was identified as a rate-limiting step. Another *A. oryzae* strain (AM1) has been able to remove 57% of 10 mM glyphosate within 13 days. The fungal strain used the pesticide contaminant as a source of nitrogen and phosphorous [71]. More importantly, it showed the capability to maintain itself along the native soil mycota, while both being negatively impacted by the glyphosate in soil microcosmos assay.

Abraham et al. [72] reported that *Aspergillus sojae* biodegraded 500 mg·L^−1^ of monocrotophos as a sole carbon source in three days. It was outperformed by the strain of *A. niger* in a previous study by Abraham and Reddy [73], which decreased the monocrotophos concentration below the detection limit within a day, while Bhalerao and Puranik [74] reported *A. oryzae* ARIFCC 1054 strain’s ability to degrade this organophosphorus, nonspecific systemic insecticide and acaricide by 70% within the first 50 h and completely in a week.

## 4. Biotransformation of Pesticide Belonging to Some Other Chemical Groups by *Aspergillus* and *Penicillium* Species

The biodegradation of three pesticides of the different chemical classes, difenoconazole, pendimethalin, and terbuthylazine, by *Penicillium brevicompactum* and *A. oryzae* was studied by Pinto et al. [75]. After an 8-day incubation, 99% of pendimethalin was degraded by both fungal strains. Fungi *A. oryzae* and *P. brevicompactum* were capable of degrading 88% and 92.7% of difenoconazole, while lower removal percentages were exhibited with terbuthylazine approximating 78% and 71%, respectively. More importantly, the authors have highlighted adsorption as a potential mechanism of pesticide removal, characterized by the fast initial removal rates, which may be an efficient mechanism utilized by the fungus to block the xenobiotic uptake.

Ahmad et al. [76] studied the biodegradation of a triazole herbicide, thiencarbazone methyl, by fungal strains of *A. niger*, *A. terreus*, *A. flavus*, *A. fumigatus* and *P. chrysogenum* isolated from soils. All fungal strains degraded pesticide efficaciously over the span of 42 days, particularly *A. terreus* was well-suited for thiencarbazone methyl degradation with a 98% degradation rate, followed by *P. chrysogenum* (95%). The lowest biodegradation efficiency was observed for *A. fumigatus* (74%). In a fungal-free control, hydrolytic reduction of thiencarbazone methyl took place and an 11% conversion of its initial 10 mg·L^−1^ was observed.

Derbalah et al. [77] reported that approximately 93% of the initial famoxadone concentration was degraded within four weeks by strains of *A. niger* EB2 and *Penicillium* sp. EB3 and the negligible spontaneous degradation was observed in control experiments. The bioassay conducted with *Alternaria solani* using the spent medium with degradation products collected at the end of famoxadone treatment showed only slight antifungal activity, indicated by 5% growth inhibition of *A. solani*.

The biodegradation of a phenylpryazole insecticide, fipronil, has been studied by Gajendiran and Abraham [78]. They isolated fipronil tolerant strain of *Aspergillus glaucus* that has shown an exceptional ability to transform it into metabolite fipronil sulfone. They also suggested the predominant involvement of manganese peroxidase in insecticide degradation.

Chang et al. [79] isolated *P. oxalicum* from an activated sludge, which performed hydrolytic and reductive dichlorination of metolachlor, a persistent chloroacetamide herbicide. Its initial 50 mg·L^−1^ concentration was degraded by 88.6% at the end of 16-day cultivation by the fungus. In addition, the enzymatic dechlorination coupled with hydroxylation, N-dealkylation, and breaking of amide linkage was reported to be responsible for the degradation of metolachlor by the homogenized crude extract of fungus *A. flavus* [80].

## 5. Concluding Remarks

Since filamentous fungi of *Penicillium* and *Aspergillus* genera can colonize very diverse niches, and Ascomycota seems to be the dominant phylum within the microbial group in various contaminated substrates, they possess great potential in the remediation of pesticide-contaminated sites. Our review highlights the fungal immense conversion of various pesticides into intermediate metabolites and/or their rapid mineralization within a few days or in couple of weeks. Different species can remove the pesticides at different rates, and to various extents; however, the fungal ability to resist high concentrations of pesticides is almost unparalleled compared to other microbial groups. Their performance may be further improved by applying indigenous strains isolated from pesticide-contaminated soils and sediments. Although this review primarily focuses on the application of batch fungal cultures, we should note that the successful development of various molecular techniques in mycoremediation allows the application of immobilized fungal enzymes in the bioremediation of soils contaminated with persistent and hazardous pesticides. However, there is still a long way to get to the real application under field conditions, since most available research reports focus on laboratory-scaled experiments under carefully regulated and optimized conditions. As shown in this review, among all listed studies, the experiments with soil microcosmos are rare. Furthermore, the available information on molecular machinery, which is a key aspect for a proper molecular engineering of the reviewed process, is insufficient or completely missing regarding the biodegradation of organochlorine and organophosphorus pesticides by fungi belonging to *Penicillium* and *Aspergillus* genera. Moreover, the more comprehensive research on cooperative biodegradation between soil aspergilli, penicillia and bacteria is needed, since it is expected that it is a synergistic relationship where the fungi can transform pesticides to metabolites that are more easily degraded by bacteria. Thus, the construction of such consortia is critical for taking the full advantage of the extraordinary metabolic diversity provided by fungi in the biodegradation of pesticides at contaminated sites. All the listed challenges should be approached unapologetically and with a high-risk high gains attitude in future research with the ultimate goal to remedy pesticide-contaminated soil, waters, and sediments without generating waste and toxins that are harmful to the environment.

## Figures and Tables

**Table 1:** Chemical structure of pesticides experimentally tested for biodegradation by fungal strains of *Aspergillus* and *Penicillium*.

Pesticide Name	Function	Chemical Structure
lindane	insecticide, acaricide and rodenticide	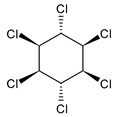
endosulfan	insecticide and acaricide	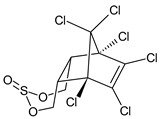
dieldrin	insecticide	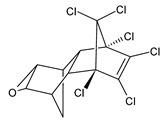
chlorpyrifos	insecticide	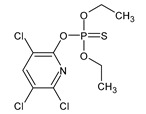
methyl parathion	insecticide	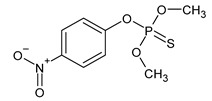
profenofos	insecticide	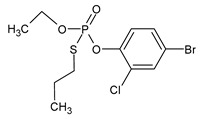
dimethoate	insecticide and acaricide	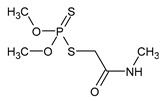
methamidophos	insecticide	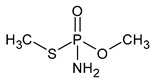
malathion	insecticide and acaricide	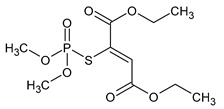
cyolan (phosfolan)	insecticide	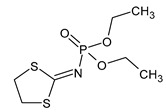
glyphosate	herbicide	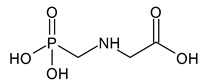

**Table 2:** Cultivation conditions and reported performances of filamentous fungi belonging to the genera *Aspergillus* and *Penicillium* in the biodegradation of organochlorine pesticides.

Fungal Strain	Degradation Efficiency	Cultivation Conditions	Degradation Products	Reference
*Penicillium* sp. CHE23	94.8%	modified mineral salt medium with ~57 mg·L^−1^ of **endosulfan** as carbon source incubated for 144 h at 30 °C and 150 rpm	not reported	Romero-Aguilar et al. [31]
*Aspergillus niger*	complete degradation	Czapek–Dox broth with 350 mg·L^−1^ of β-**endosulfan** source incubated for 120 h at 30 °C and 120 rpm	endosulfan sulfate being most persistent metabolite; after 120 h, complete mineralization was suggested	Bhalerao and Puranik [32]
*Aspergillus niger* ARIFCC 1053	complete degradation	Czapek–Dox broth with 1000 mg·L^−1^ of technical-grade **endosulfan** incubated for 7 days at 30 °C and 120 rpm	complete mineralization	Tejomyee [33]
*Aspergillus niger*	98.6%	Czapek–Dox broth with 15.4 µg·g^−1^ of technical-grade **endosulfan** incubated for 15 days at 30 °C and intermittent shaking	not reported	Mukherjee and Gopal [34]
*Aspergillus terreus*, (*Cladosporium oxysporum*)	91.5%, (89%)	potato dextrose broth with 1.89 µg·g^−1^ of technical-grade **endosulfan** incubated for 15 days at 25 °C	trace concentrations of endosulfan sulfate were detected during incubation; no end products were reported	Mukherjee and Mittal [35]
*Aspergillus terricola*, *Aspergillus terreus*, (*Chaetosartorya stromatoides*)	~90%	non-sulfur medium enriched with 100 mg·L^−1^ of α or β **endosulfan** isomers incubated for 12 days at 30 °C and 150 rpm (pH 6)	major metabolic products being endosulfan diol and endosulfan ether	Hussain et al. [36]
*Aspergillus niger* AE	complete degradation (0.1% endosulfan), and 76% (0.5% endosulfan)	Czapek–Dox broth spiked up to 5000 mg·L^−1^ (0.5%) of **endosulfan** incubated for 8 days at 30 °C and 180 rpm (optimum at pH 4)	not reported	Mukhtar et al. [37]
*Aspergillus niger*, *Aspergillus flavus*, *Penicillium chrysogenum*	77% (AN), 72% (AF), 69% (PC)	potato dextrose broth with 10 mg·L^−1^ of **endosulfan** incubated for 35 days at 29 °C	desulphurized transformants of endosulfan, while chlorine atoms remained imperforated	Ahmad [38]
*Aspergillus sydoni*	95% (α isomer), 97% (β isomer)	Czapek–Dox broth with 100 mg·L^−1^ of α or β **endosulfan** isomers incubated for 18 days at 30 °C and 150 rpm	major metabolic products being endosulfan sulfate	Goswami et al. [39]
*Aspergillus tamarii* JAS9, (*Botryosphaerialaricina* JAS6)	kinetic analysis shows that 50% of α and β endosulfan isomers were degraded in 1.7 and 2.2 days by JAS9, respectively (4.2 days for 50% reduction of β endosulfan by JAS6)	M1 medium with 1000 mg·L^−1^ of technical-grade **endosulfan** as carbon source incubated for 10 days at 30 °C and 120 rpm	not reported	Silambarasan and Abraham [40]
fungal consortium (*Botryosphaeria laricina* JAS6, *Aspergillus tamarii* JAS9 and *Lasiodiplodia* sp. JAS12)	complete degradation (a 50% degradation calculated on the 3rd day of incubation)	M1 medium with 1000 mg·L^−1^ of technical-grade **endosulfan** as carbon source incubated for 120 h at 30 °C and 120 rpm	complete mineralization	Abraham and Silambarasan [41]
*Penicillium camemberti*	70%	acetate-free basal medium supplemented with 1 mM **lindane** incubated for 120 h at 25 °C and 80 rpm (pH 5)	not reported	Taşeli [42]
*Aspergillus fumigatus*	complete degradation	**lindane** initial concentration is not indicated; medium (Sabouraud dextrose broth or Nutrient broth) incubated for 5 days at 25 °C	not reported	Kumaravel et al. [43]
*Penicillium miczynskii* CBMAI 93	90%	culture medium of artificial salt water supplemented with 50 mg·L^−1^ of **dieldrin** incubated for 14 days at 32 °C and 130 rpm	no intermediate degradation products were detected, suggesting dieldrin mineralization of conjugation	Birolli et al. [44]

**Table 3:** Cultivation conditions and reported performances of filamentous fungi belonging to the genera *Aspergillus* and *Penicillium* in the biodegradation of organophosphorus pesticides.

Fungal Strain	Degradation Efficiency	Cultivation Conditions	Degradation Products	Reference
*Aspergillus sydowii* CBMAI 935	32%, 80% and 52% of chlorpyrifos, methyl parathion, and profenofos, respectively	2% malt liquid medium with 50 mg·L^−1^ of **chlorpyrifos, methyl parathion** or **profenofos** incubated for 30 days at 32 °C and 130 rpm	chlorpyrifos degradation resulted in tetraethyl dithiodi- phosphate and 2,3,5-trichloro-6-methoxypyridine; methyl parathion hydrloyzed into methylated phosphate and phosphorothioates, and 1-methoxy-4-nitrobenzen; profenofos degraded into 4-bromo-2-chloro-1-methoxybenzen and *O*,*O*-diethyl-*S*-proyl phosphorothioates	Soares et al. [50]
*Aspergillus sydowii* CBMAI 935, *Penicillium decaturense* CBMAI 1234	80%	liquid mineral medium supplemented with KNO_3_ and 100 mg·L^−1^ of **methyl parathion** incubated for 30 days at 32 °C and 130 rpm	*p*-nitrophenol	Alvarenga et al. [51]
*Aspergillus niger* AN400	2% (glucose-free treatment, initial concentration of methyl parathion was 19.1 mg·L^−1^), 43% (glucose-treated medium, initial concentration of methyl parathion 24.9 mg·L^−1^)	glucose-free or glucose treated distilled water supplemented with Vishniac solution and up to 24.9 mg·L^−1^ of **methyl parathion** incubated for 27 days at 30 °C and 200 rpm	not reported	Marinho et al. [52]
*Penicillium citrinum*, *(Fusarium proliferatum)*	complete degradation (the biotic control had the same degradation efficiency)	3% malt liquid medium with 30 mg·L^−1^ of **methyl parathion** incubated for 30 days at 32 °C and 130 rpm	not reported	Rodrigues et al. [53]
*Aspergillus niger* MRU01	70%, 54%, 58%, and 68% of malathion, parathion, chlorpyrifos and dimethoate, respectively	Czapek–Dox broth spiked with 500, 470, 260 and 680 μmol·L^−1^ (0.5%) of **malathion**, **parathion**, **chlorpyrifos,** and **dimethoate**, respectively, incubated for 5 days at 26 °C and 120 rpm (optimum at pH 4)	not reported	Mohapatra et al. [54]
*Aspergillus flavus*	complete degradation	mineral salt medium supplemented with 5 mg·L^−1^ of **malathion** incubated for 36 days at 30 °C (pH 7) on a rotatory shaker (optimized conditions)	not reported	Derbalah et al. [55]
*Aspergillus* sp. F1	over 90% (89% at an inlet load of 180 mg·L^−1·^d^−1^)	bioreactor supplemented with 300 mg·L^−1^ of **chlorpyrifos** as the sole carbon source incubated at 28 °C (pH 7) with dissolved oxygen concentration of 5.8 mg·L^−1^ (optimized conditions)	not reported	Yadav et al. [56]
*Aspergillus fumigatus*	99%	potato dextrose broth supplemented with **chlorpyrifos** (10%) incubated for 9 days at 25 °C (pH 7) and 180 rpm	not reported	Anggreini et al. [57]
*Aspergillus fumigatus*	95.9%	potato dextrose broth with **chlorpyrifos** (1.5%) incubated for 5 days at 25 °C (pH 7) and 180 rpm	not reported	Anggreini et al. [58]
*Aspergillus terreus* JAS1	complete degradation (after 24 h)	M1 medium supplemented with 300 mg·L^−1^ of **chlorpyrifos** as the sole carbon source incubated for 96 h at 30 °C and 120 rpm	3,5,6-trichloropyridin-2-ol that was completely degraded after 48 h; no other metabolites were reported	Silambarasan and Abraham [59]
*Aspergillus oryzae* strains AM1 and AM2	73% (AM1), 50% (AM2)	Czapek–Dox broth spiked with 20 mg·L^−1^ of **chlorpyrifos** incubated for 30 days at 25 °C and 60 rpm (optimum at pH 4)	not reported	Barberis et al. [60]
*Aspergillus viridinutans*, *Penicillium implicatum*	44.6% (*A. viridinutans*), 16.2% (*P. implicatum*)	potato dextrose broth with 20 mg·L^−1^ of **chlorpyrifos** incubated for 14 days at 28 °C	not reported; high losses of chlorpyrifos from culture medium were due to abiotic hydrolysis	Abdel-Wareth and Abd El-Hamid [61]
*Aspergillus niger*, *(Trichoderma viride)*	72.3%, (95.7%)	Czapek–Dox broth spiked with 1.25 mg·L^−1^ of **chlorpyrifos** incubated for 14 days at 30 °C and intermittent shaking (pH 6.8)	not reported; high losses of chlorpyrifos from culture medium were due to abiotic hydrolysis	Mukherjee and Gopal [62]
*Penicillium citrinum*, *Aspergillus niger*, *Aspergillus oryzae*	25.9% (*P. citrinum*), 64% (*A. niger*), 50.8% (*A. oryzae*)	Burkes mineral broth with 10 mg·L^−1^ of **chlorpyrifos** incubated for 15 days at 27 °C without shaking (pH 7.2)	not reported; high losses of chlorpyrifos from culture medium were due to abiotic hydrolysis	Abd-Alrahman and Mostafa [63]
*Aspergillus niger*	~80%	soil extract medium with 10 mg·L^−1^ of **chlorpyrifos** incubated for 30 days at 25 °C and 60 rpm	3,5,6-trichloro-2-pyridinol were detected below the concentration of 1 mg·L^−1^	Karas et al. [22]
*Aspergillus fumigatus*, *A. flavus*, *A. niger*, *A. ochraceus*, *A. tamarii*, *A. terreus*, *Penicillium chrysogenum*, *P. brevicompactum*, *P. citrinum*, *P. funiculosum*	phosphor mineralization efficiencies ranged from 4 to 46% (Cyolan), from 9.5 to 26.8% (Malathion), and from 2.3 to 6.7% (Dursban)	Czapek–Dox broth spiked with 100 mg·L^−1^ of **cyolan**, **malathion**, and **chlorpyrifos** incubated for 35 days at 28 °C without shaking	not reported; media and biomass were analyzed for phosphor and sulfur that mineralized from the degradation of insecticide	Omar [64]
*Penicillium oxalicum* ZHJ6	complete degradation	mineral salt medium with 1% glucose supplemented with 1 mg·L^−1^ of **methamidophos** as sole nitrogen source incubated for 12 days at 25 °C and pH of 5.0 (most favorable conditions)	inorganic phosphor, CH_3_SH, and CH_3_OH are hypothesized being formed	Zhao et al. [65]
*Aspergillus oryzae* A-F02	~13.7%	fermentation medium with 1.5 g·L^−1^ of **glyphosate** incubated for 144 h at 30 °C and 150 rpm	aminomethylphosphonic acid and methylamine, the latter being further degraded	Fu et al. [66]

## Data Availability

Not applicable.
